# Evaluation of the Thermal Inactivation of a *Salmonella* Serotype Oranienburg Strain During Cocoa Roasting at Conditions Relevant to the Fine Chocolate Industry

**DOI:** 10.3389/fmicb.2021.576337

**Published:** 2021-03-08

**Authors:** Runan Yan, Gabriella Pinto, Rebecca Taylor-Roseman, Karen Cogan, Greg D’Alesandre, Jasna Kovac

**Affiliations:** ^1^Department of Food Science, The Pennsylvania State University, University Park, PA, United States; ^2^Dandelion Chocolate Inc., San Francisco, CA, United States

**Keywords:** *Salmonella*, cocoa beans, roasting, microbial inactivation, food safety

## Abstract

Cocoa roasting produces and enhances distinct flavor of chocolate and acts as a critical control point for inactivation of foodborne pathogens in chocolate production. In this study, the inactivation kinetics of *Salmonella enterica subsp. enterica* serotype Oranienburg strain was assessed on whole cocoa beans using roasting protocols relevant to the fine chocolate industry. Beans were inoculated with 10^7^–10^8^ log_10_ CFU/bean of *Salmonella* Oranienburg and roasted at 100–150°C for 2–100 min. A greater than 5 log_10_ reduction of *S.* Oranienburg was experimentally achieved after 10-min roasting at 150°C. Data were fitted using log-linear and Weibull models. The log-linear models indicated that the roasting times (*D*) needed to achieve a decimal reduction of *Salmonella* at 100, 110, 115, 120, 130, and 140°C were 33.34, 18.57, 12.92, 10.50, 4.20, and 1.90 min, respectively. A Weibull model indicated a decrease in the *Salmonella* inactivation rate over time (*β* < 1). Statistical analysis indicated that the Weibull model fitted the data better compared to a log-linear model. These data demonstrate the efficacy of cocoa roasting in inactivation of *Salmonella* and may be used to guide food safety decision-making.

## Introduction

*Salmonella enterica subsp. enterica* serotypes have been associated with foodborne outbreaks linked to low moisture food products such as chocolate (Craven et al., 1975; Hockin et al., 1989; Werber et al., 2005), cereals (Russo et al., 2013), nuts (Kirk et al., 2004; Bedard et al., 2014), nut butters (CDC, 2009; Sheth et al., 2011), flour (McCallum et al., 2013), and spices (Van Doren et al., 2013; Jernberg et al., 2015). These foods often have water activity (*a_w_*) below 0.85, which prohibits the growth of most bacteria, including *Salmonella* (Young et al., 2015). However, exposure of *Salmonella* cells to sublethal stresses, such as desiccation, could potentially lead to its adaptation and increased resistance to subsequent heat treatment (Baird-Parker et al., 1970; Goepfert et al., 1970; Keller et al., 2012; Villa-Rojas et al., 2013; Jin et al., 2018). Microbial cells may survive in the desiccated state in low moisture foods during extended storage (Tamminga et al., 1976; Farakos et al., 2013; Beuchat et al., 2017) and resume active metabolism and growth upon returning to a favorable environment. In addition to the previous exposure to stresses, the ability of individual microbial strains to resist heat treatment in low *a_w_* foods also varies (Izurieta and Komitopoulou, 2012; Alvarenga et al., 2018), which could potentially be to some extent attributed to microbial genetic background (Mercer et al., 2015, 2017). The effectiveness of thermal inactivation can be further influenced by the food composition, such as protein (Jin et al., 2018) and fat content (Kataoka et al., 2014; Li et al., 2014), which alter the food microenvironment and may provide a protective effect against heat treatment. When generating data to support food safety decision-making, it is therefore important to evaluate the thermal inactivation kinetics using relevant pathogen strains, at relevant conditions and food matrices that mimic real-life scenarios as closely as possible.

Thermal inactivation data can be modeled using a variety of thermal inactivation models. These include log-linear and Weibull models that have been developed to study the inactivation kinetics of microorganisms in different food matrices (Jin et al., 2018). These models utilize experimentally obtained data to predict the thermal treatment conditions required to achieve the desired level of microbial inactivation. The most commonly applied log-linear model assumes that the inactivation of microorganisms follows the first-order kinetics (Bigelow and Esty, 1920; Bigelow, 1921). A mechanistic explanation for the log-linear model is that the death of vegetative cells is caused by inactivation of enzymes critical for microbial survival, and that the enzyme inactivation is governed by the first-order kinetics (Lencki et al., 1992). Such models implicitly assume that each bacterial cell has an equal probability of inactivation under a given treatment, and hence do not model a microbial population response realistically (Boekel, 2002). An alternative, Weibull model, models the inactivation of microorganisms as a probabilistic rather than a deterministic process. It considers a variable range of heat resistance among individual cells within a microbial population, as well as the heterogeneity of the thermal treatment conditions (Boekel, 2002; Mafart et al., 2002). Although the log-linear model is widely used due to the simplicity and intuitive nature of the first-order kinetics, the Weibull model can more accurately model thermal inactivation, and therefore provide more realistic and valuable data to support food safety decision-making.

Cocoa roasting is an important flavor and aroma development process, as well as a critical food safety step in chocolate production (Counet et al., 2004; Hurst et al., 2011). Roasting facilitates the development of desired chocolate flavor that results from interactions between amino acids and sugars through Maillard reaction and Strecker degradation (Ramli et al., 2006; Sacchetti et al., 2016). Fine chocolate producers therefore utilize roasting procedure to achieve unique sensory properties characteristic of products derived from single-origin cocoa (Beckett et al., 2017). Generally there is a positive correlation between the flavor quality and the roasting temperature (Oracz and Nebesny, 2016); however, over-roasting (beyond 160°C) can result in the development of undesired off-flavors and burnt taste. The fine chocolate industry therefore often carries out roasting at temperatures lower than those commonly utilized in mass chocolate production and roasting is often carried out for shorter periods of time. Roasting protocols can vary from 110 to 160°C (usually 120–140°C) for as short as 5 min or as long as 120 min (usually 10–35 min; Aprotosoaie et al., 2016). Lower temperatures (90–110°C) could also be used for cocoa roasting and have been found to increase the acceptance by consumers (Rocha et al., 2017). While cocoa roasting parameters have been extensively studied from the sensory perspective (Ramli et al., 2006; Hurst et al., 2011; Farah et al., 2012; Krysiak et al., 2013; Zzaman and Yang, 2014; Rocha et al., 2017; Zzaman et al., 2017), the efficacy of roasting in terms of enteric foodborne pathogen inactivation is yet to be evaluated. Hence, we aimed to evaluate *Salmonella* inactivation at a range of temperatures that may be relevant to fine chocolate producers to assist in the selection of cocoa roasting condition from a food safety perspective.

We focused on the characterization of the thermal inactivation of *Salmonella enterica subsp. enterica* serotype Oranienburg strain on seeds of *Theobroma cacao* (cocoa beans) that are typically grown approximately 10° north and south of the equator in rain forests (Kole, 2011). Traditionally, fresh cocoa beans are collected from ripe cacao pods approximately 7–10 days after pod harvesting. The collected beans are then subjected to natural fermentation to produce flavor precursors that are needed for a quality chocolate product. The fermented beans are dried naturally by sun or mechanically to reduce the moisture content to approximately 2–5% (Aremu et al., 1995). Cacao growers often carry out sun-drying because it results in lower acidity, astringency, and bitterness of dried cocoa beans, which increases their value on the fine chocolate market. However, this practice, especially if conducted outdoors, increases the chances of fecal contamination and introduction of enteric foodborne pathogens *via* animal intrusion (Akanele et al., 2016). The risk of contamination with enteric foodborne pathogens such as *Salmonella* emphasizes the importance of cocoa roasting, which is a critical food safety control point in the process of chocolate production (ICMSF, International Commission on Microbiological Specifications for Foods and Swanson, 2011).

To date, only two studies have assessed thermal inactivation kinetics of *Salmonella* during cocoa roasting (Izurieta and Komitopoulou, 2012; Nascimento et al., 2012). Furthermore, there is a lack of available data characterizing the thermal inactivation of *Salmonella* at lower temperatures (below 160°C) and shorter roasting times, which are relevant to the fine chocolate industry, which utilizes cocoa roasting also as a critical flavor compound generating step. We therefore investigated the thermal inactivation of *Salmonella* Oranienburg strain on cocoa beans roasted at temperatures below 160°C to generate data that may guide food safety decision-making in the fine chocolate industry. We applied log-linear and Weibull models to model the thermal inactivation of *S.* Oranienburg strain at different cocoa roasting conditions tested. The main objectives of this study were to (1) assess the effectiveness of the selected roasting treatments in inactivation of the selected model *S.* Oranienburg strain, and (2) compare the performance of log-linear and Weibull models in modeling the thermal inactivation of the selected *Salmonella* strain during cocoa roasting.

## Materials and Methods

### Bacterial Strain

A strain of *Salmonella enterica subsp. enterica* serotype Oranienburg (FSL R9-6137) was obtained from the Food Microbe Tracker isolate collection (Vangay et al., 2013). The strain FSL R9-6137 originates from a human and was provided to the Food Microbe Tracker isolate collection by the New York Department of Health. Culture of the isolate was preserved and stored at −80°C in a brain heart infusion broth (BHI broth, BD Difco) supplemented with 25% (*v*/*v*) glycerol.

### Preparation of Cocoa Beans

Cocoa beans imported from Bolivia (Nwanosike et al., 2019) were obtained from Dandelion Inc. (San Francisco, CA). Cocoa beans were autoclaved twice at 121°C for 30 min to inactivate natural contaminants present on the cocoa beans. Autoclaved beans were then air-dried in a BSL-2 cabinet for up to 2 h. The water activity of beans (*N* = 10) was measured before autoclaving and after autoclaving followed by a 2-h drying in a biosafety cabinet at a room temperature. Water activity was measured using a bench top water activity meter AquaLab Series 4TE (Decagon Devices Inc., Pullman, WA) with a ±0.003 precision at 25°C. Autoclaved and dried beans were used in all experiments reported in this study. A negative control consisting of uninoculated autoclaved dried beans was included in each experiment to ensure absence of background microbiota and cross-contamination. Only cocoa beans with a weight of 1.0 ± 0.2 g were used in the experiments to minimize the potential variability in results caused by the non-uniformity of water migration from the core to the shell of the bean during roasting.

### Preparation of an Inoculum

*Salmonella* strain was sourced from a glycerol stock stored at −80°C and was streaked onto xylose lysine deoxycholate (XLD) agar (Hardy Diagnostics, Santa Maria, CA) to control for culture purity. Streaked XLD plates were incubated at 35°C for 24 ± 1 h. After incubation, three well-isolated single colonies were streaked in lawns onto three individual brain heart infusion agar plates (BHI agar; BD Difco) to obtain cultures for three biological replicates. After 24 h of incubation at 35°C, bacterial lawns were flooded with 3–5 ml of sterile phosphate buffer saline (PBS, containing 137 mM NaCl, 2.7 mM KCl, 8 mM Na_2_HPO_4,_ and 2 mM KH_2_PO_4_, pH 7.4) pre-warmed to room temperature. The *Salmonella* biomass was harvested from the BHI agar surface by scraping with a sterile L-shaped spreader. The suspended biomass was then aseptically transferred into a sterile conical tube using a serological pipette, vortexed, and diluted 1:5 in PBS pre-warmed to room temperature (Komitopoulou and Peñaloza, 2009). The concentration of the diluted culture suspension was determined by measuring optical density at 600 nm using a Bio Photometer (Eppendorf, Germany). The culture concentration in CFU/ml was determined based on a previously determined OD-CFU/ml standard curve developed for *Salmonella*.

### Inoculation of Cocoa Beans

Each time-temperature condition was tested in a minimum of three biological test replicates with corresponding three biological positive control replicates, where each cocoa bean represented one biological replicate. Each biological replicate was quantified in three technical replicates. Individual beans were inoculated at 10^7^–10^8^ *Salmonella* cells per bean. Each sterilized cocoa bean was spot inoculated with 3–9 μl of *Salmonella* inoculum, depending on the concentration of inoculum collected from lawn plates. The water activity (*a_w_*) of whole beans both before and after inoculation of 3 and 9 μl of PBS was determined using the bench top water activity meter (AquaLab), to assess the effect of inoculation and stabilization procedures on *a_w_*. To minimize the effect of inoculation volume on the water activity of the beans and to stabilize the inoculum, each inoculated cocoa bean was stored in a covered sterile Petri dish in an air incubator at 20°C for 16 h. Stacking of plates was avoided to allow for a uniform air circulation and to prevent the accumulation of a condensate during the inoculum stabilization. For each independent experiment, an uninoculated bean was included as a negative control to confirm the absence of natural contaminants and of cross-contamination.

### Thermal Inactivation of Salmonella in Cocoa Roasting Procedures

Sterile cocoa beans were inoculated and inoculum stabilized as described above. Cocoa beans were then individually transferred into aluminum weighing dishes using sterile forceps. The aluminum weighing dishes containing cocoa beans were then transferred into a pre-heated convection oven (Binder FD 53-UL or VWR 1330FMS) and were heated at different time-temperature combinations. The temperature of air flowing above the cocoa beans was monitored by a thermometer (Fluke corporation, WA), and recorded in 1-min intervals. The treatment started when the air temperature above the beans reached the target temperature. The measured temperature variation was within ±1°C of the target treatment temperature. The relative humidity within the oven during roasting was 0.0%, as determined using VAIS-Indigo 201 Vaisala humidity probe after reaching a target temperature. After completed roasting (e.g., incubation at a target temperature for a target period of time), the aluminum weighing dishes with beans were removed from the oven, and each bean was immediately transferred into a 7-oz sterile homogenization bag (Weber Scientific, NJ) pre-filled with 9 ml of cold sterile PBS. The beans were then homogenized at 230 rpm for 1 min using a paddle blender (Seward Ltd., England), followed by hand massaging the homogenate to enhance the recovery of *Salmonella* cells from bean debris to the buffer. The homogenized suspensions were then immediately serially diluted in PBS and plated onto BHI agar plates in triplicates to minimize the exposure to cocoa phenolic compounds that could potentially inhibit heat-injured *Salmonella* cells. Inoculated BHI plates were incubated at 35°C for 24 ± 2 h. Three positive controls were prepared following the same inoculation, stabilization, homogenization, and enumeration protocol and were quantified to determine the initial *Salmonella* load before roasting. One negative control was roasted and enumerated in each experiment, following the same procedure. The average *Salmonella* reduction for each treatment condition was calculated by subtracting the average quantities of *Salmonella* on roasted beans from the average quantities of *Salmonella* on positive controls.

### Modeling of Salmonella Inactivation Using Log-Linear and Weibull Models

The log-linear (Bigelow, 1921) and the Weibull (Boekel, 2002; Mafart et al., 2002) models were used to describe the inactivation of *Salmonella* during cocoa roasting procedures. The most widely accepted log-linear model (Bigelow, 1921) assumes that the inactivation of bacterial vegetative cells follows the first-order kinetics:

$$\log N_{t}=\log N_{0}-\frac{t}{D}$$

where *N_t_* represents the *Salmonella* load (CFU/bean) after thermal treatment for time t, *N_0_* represents the initial *Salmonella* load (CFU/bean) at time 0, and *D* is the decimal reduction time (*D* value). To describe the temperature dependence of *D* values, a secondary log-linear model was applied, where the *z_T_* is defined as the increase of temperature that results in a reduction of a *D* value by a factor of 10 (Bigelow, 1921). The *z_T_* was calculated using the following equation:

$$z_{T}=\frac{T_{2}-T_{1}}{\log\left(D_{T1}\right)-\log\left(D_{T2}\right)}$$

where *D_T1_* and *D_T2_* indicate the *D* values at temperature *T_1_* and *T_2_* determined using the log-linear model.

To account for cases where the thermal death curves of bacteria do not follow the first-order kinetics, the Weibull regression model (Boekel, 2002; Mafart et al., 2002) was used as an alternative approach to evaluate the inactivation of *Salmonella* during the roasting procedure, using the following equation:

$$\log N_{t}=\log N_{0}-{\left(\frac{t}{\delta }\right)}^{\beta }$$

where *δ* is defined as the time required for the first decimal reduction in a process, and the fitting parameter *β* defines the shape of the curve. The shape parameter *β* describes whether the response curve is linear (*β* = 1) or nonlinear (*β* ≠ 1) with a decreasing (*β* < 1) or increasing (*β* > 1) inactivation rate with time. Parameters *δ* and *β* were estimated for individual roasting temperature.

Similar to the *D* value described before, the temperature dependence of the scale parameter *δ* was modeled *via* an exponential relationship, and thus was considered as an alternative to the *D* value (Boekel, 2002). The *z'* value for the Weibull model, which is analogous to the *z_T_* obtained using a log-linear model, was defined using the following equation (Boekel, 2002):

$$z^{\prime }=\frac{T_{1}-T_{2}}{\log\;\delta_{T2}-\log\;\delta_{T1}}$$

where *δ_T1_* and *δ_T2_* are the time required to achieve the first-log reduction at temperature *T_1_* and *T_2_* determined using the Weibull model.

For the Weibull model, the time required for a desired amount of microbial reduction, *t_D_*, was calculated using the shape and scale parameters as shown below:

$$t_{D}=\delta {\left(-\log\left(10^{-D}\right)\right)}^{\frac{1}{\beta }}$$

where *D* represents the desired decimal reduction of a given microbial population. For example, if *D* = 1, the calculated *t_D_* would be the required roasting time for a 1-log_10_ *Salmonella* reduction predicted by the Weibull model. Likewise, if *D* = 5, the calculated *t_D_* would then be the roasting time required to achieve a 5-log_10_ reduction.

### Statistical Analysis

The difference in *Salmonella* reduction caused by temperature was considered significant if the *p* value was equal or lower than 0.05. Thermal inactivation kinetics during cocoa roasting was analyzed using log-linear and Weibull models using the “nls” function from package “nlstools” (Baty et al., 2015) by nonlinear least squares methodology using Gauss-Newton algorithm in R (R Core Team, 2019). Model’s goodness-of-fit was evaluated based on relative standard error (RSE) of estimated parameters and the root mean square of residuals (RMSE) of the model. The relative quality of both log-linear and Weibull model was evaluated based on the out-of-sample prediction error by calculating two estimators: Akaike information criterion (AIC) and Bayesian information criterion (BIC) values. A better model fit is indicated by a smaller AIC or BIC value. A significantly higher goodness-of-fit was determined using ANOVA when a *p* value was smaller than 0.05.

## Results and Discussion

Due to the fact that a serotype *S.* Oranienburg, albeit not this specific strain of *S.* Oranienburg, has been associated with an international outbreak linked with chocolate products in 2005 (Werber et al., 2005), we selected a strain FSL R9-6137 of this serotype for cocoa roasting experiments. In the present study, we chose to use a convection oven and intact cocoa beans to better mimic the conditions in an industrial roaster and produce the results that are more directly relevant to the fine chocolate producers compared to experiments in which water activity is controlled throughout roasting experiments.

### *D* and *z* Values Were Determined Using Both Log-Linear and Weibull Models

The thermal inactivation kinetics of *S.* Oranienburg during cocoa roasting was evaluated at the following temperatures: 100, 110, 115, 120, 130, 140, and 150°C. The hold times at these temperatures varied from 2 to 100 min. The temperature come-up time (i.e., time from placement of the beans into the oven until the temperature rose back to the target temperature) in the oven ranged from 45 to 100 s (data not shown). The average *a_w_* of beans prior to autoclaving to inactivate background microbiota was 0.4607 ± 0.0188 (*N* = 10). After autoclaving (before inoculation), the average *a_w_* of beans was 0.474 ± 0.007 (*N* = 10), which was not significantly different compared to *a_w_* before autoclaving (*p* = 0.5621). Eliminating background microbiota from cocoa beans could potentially result in underestimated decimal reduction times, if the background microbiota would have significantly increased the heat resistance of *Salmonella*. However, there is insufficient scientific evidence to substantiate this concern.

For cocoa roasting experiments, volumes of inoculum varied between 3 and 9 μl. The average *a_w_* of beans inoculated with 3 ul inoculum was 0.475 ± 0.004 (*N* = 5) and of beans inoculated with 9 μl inoculum was 0.483 ± 0.005 (*N* = 5), demonstrating that the inoculum did not significantly change the water activity of cocoa beans after inoculation and 16-h culture stabilization (*p* = 0.105). In each independent experiment, the background microbiota on autoclaved cocoa beans was confirmed to be below the limit of detection (<1 log_10_ CFU/bean) using direct plating of negative control sample dilutions onto BHI agar plates. The reduction times achieved at different temperature-time combinations are listed in the Supplementary Table S1. The maximum average reductions of *Salmonella* at each roasting temperature were achieved after longest roasting times. At 100, 110, 115, 120, 130, and 140°C, the maximum reductions achieved were 2.80 ± 0.09, 2.87 ± 0.08, 3.51 ± 0.05, 4.36 ± 0.91, 4.62 ± 0.10, 4.98 ± 0.51 log_10_ CFU/bean, respectively. Among all cocoa roasting conditions, an average reduction above 5 log_10_ CFU/bean was experimentally achieved only when beans were roasted at 150°C for 10 min (*N* = 3). The limit of detection of the method used in this study was 1.3 log_10_ CFU/bean and the *Salmonella* log_10_ CFU/bean after 10-min roasting at 150°C was below this limit of detection. After a shorter roasting for 2 and 5 min at 150°C, the average reduction of *S.* Oranienburg strain was 1.42 ± 0.15 and 3.07 ± 0.29 log_10_ CFU/bean (*N* = 3), respectively.We initially compared the recovery of heat-treated *Salmonella* colonies on a selective (XLD) and non-selective (BHI) agars and found that approximately 0.5 log higher quantity of cells was recovered on a BHI agar, suggesting possible inhibition of heat-injured cells by selective ingredients of XLD. We therefore used BHI to recover *Salmonella* cells in all experiments reported in this study, to enhance the recovery of heat injured. *Salmonella* was recovered by incubating inoculated BHI plates without prolonged incubation (beyond 24-h).

**Figure 1:**
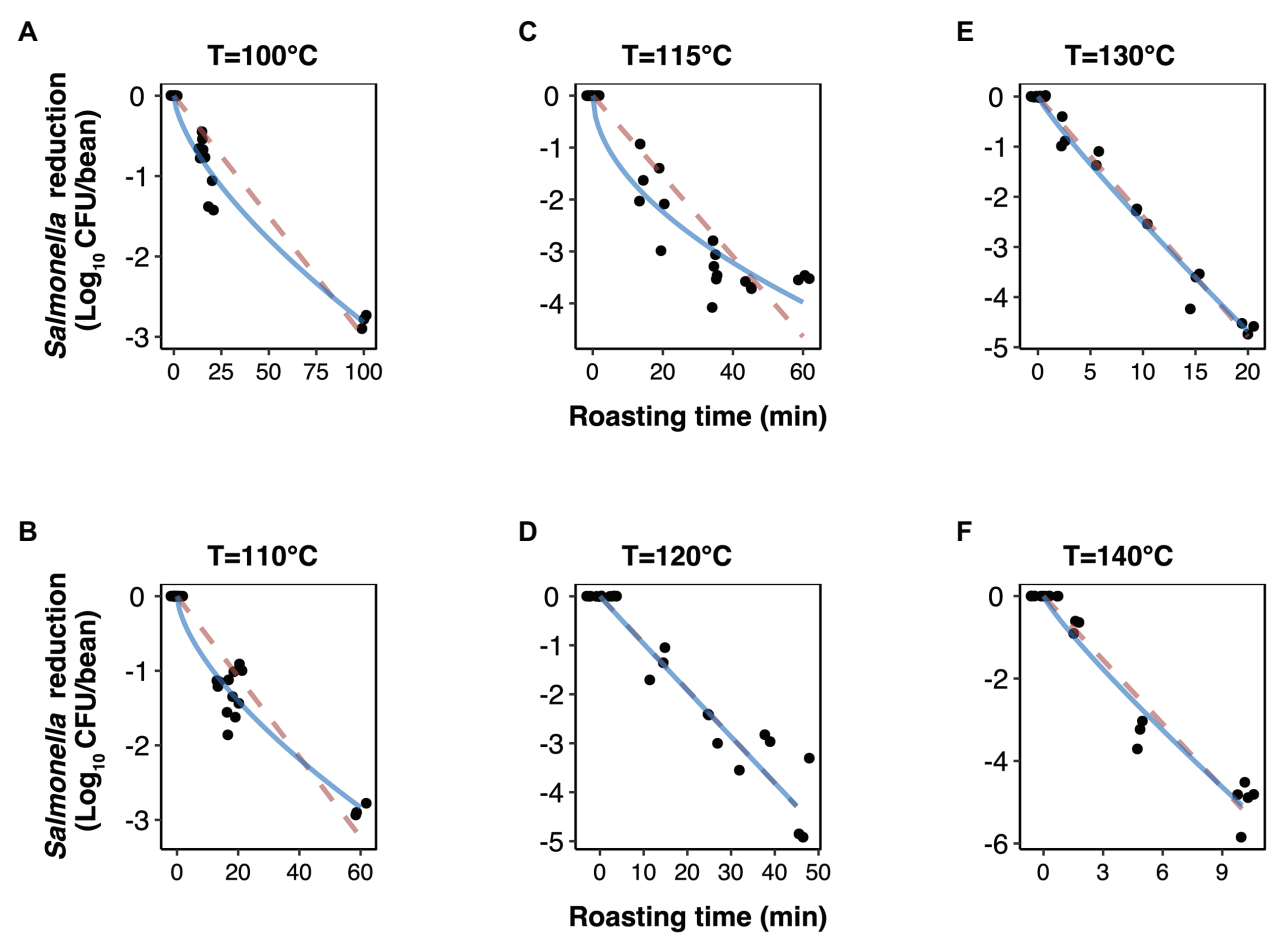
Inactivation kinetics of *Salmonella* Oranienburg strain FSL R9-6137 during cocoa roasting at different temperatures (A–F) fitted using a log-linear (dashed pink line) and a Weibull model (solid blue line). Each datum point represents the calculated reduction of *S.* Oranienburg calculated based on one biological replicate tested in three technical replicates. The plotted data points were jittered to avoid plotting them directly on top of each other. A minimum of three biological replicates were tested at each roasting condition.

The experimentally obtained inactivation data were modeled using log-linear model and the Weibull model (Figure 1). As shown in Table 1, the residual mean standard error (RMSE) of the log-linear model ranged from 0.20 to 0.52 log_10_ CFU/bean, and the RSE (%) parameter ranged from 1.74 to 5.04. The *D* values, which indicated the time needed to achieve a 1 log_10_ reduction in *S.* Oranienburg counts at a given temperature, were 33.34, 18.57, 12.92, 10.50, 4.20, and 1.90 min at 100, 110, 115, 120, 130, and 140°C, respectively. As expected, the *D* values were lower when the cocoa beans were roasted at higher temperatures, suggesting that the *S.* Oranienburg inactivation was more effective at higher roasting temperatures. Since the inactivation data at 150°C were only determined at two roasting time intervals (due to reduction below the limit of detection at longer roasting times), it was not possible to model the *D* value at this temperature. Based on the log-linear model, we determined the *z_T_* value of 32.0 ± 4.7°C, which indicated that an average increase in temperature of 32°C is required to achieve a 90% decrease in a *D* value.

**Table 1:** Comparison of the quality of log-linear and Weibull models developed to model inactivation of *S.* Oranienburg strain FSL R9-6137 at different cocoa roasting temperatures.

Temperature (°C)	Log-linear model^a^		Weibull model^a^		*P*^b^
	AIC	BIC	AIC	BIC	
100	9.47	12.06	−19.04	−15.15	1.30'10^−7^
110	17.85	21.01	−12.30	−7.55	4.38'10^−8^
115	63.14	66.47	−19.04	40.22	1.20'10^−7^
120	19.79	22.15	21.78	25.32	0.91
130	−8.58	−5.92	−11.14	−7.15	0.04
140	25.64	27.83	25.46	28.73	0.16

a AIC, Akaike information criterion; BIC, Bayesian information criterion. The relative quality of both log-linear and Weibull models was evaluated based on the out-of-sample prediction error by calculating AIC and BIC. A better model fit is indicated by a smaller AIC or BIC value.

b *P*, *p* value. A significantly higher goodness-of-fit was determined using ANOVA when a *P* value was smaller than 0.05.

In contrast to our findings, Izurieta and Komitopoulou (2012) reported a *D_T_* value of 2.56 min at 100°C for the *S.* Oranienburg strain tested in their study, and a *D_T_* value of 3.62 min at 100°C for the *Salmonella* Enteritidis strain tested in their study. Both of these *D_T_* values are substantially lower compared to *D_T_* value of 33.34 min determined in the present study. Furthermore, Nascimento et al. (2012) determined the average *D_T_* values for a cocktail of different *Salmonella* serotypes inoculated onto cocoa beans as 4.79, 3.62, 2.55, and 2.55 min at 110, 120, 130, and 140°C, respectively. In addition to the difference in food matrix and thermal treatment method used, the differences in *D_T_* values may also be attributed to various degrees of thermal resistance of different *Salmonella* strains tested, and different inoculum preparation and stabilization procedures (Nascimento et al., 2012). Lastly, the preparation of inoculum using cells recovered from bacterial lawns (used in our study), as compared to liquid broth, has been reported to significantly enhance the thermal resistance of *Salmonella* (Hildebrandt et al., 2016; Limcharoenchat et al., 2018). Bacterial cells harvested from lawns were shown to exhibit not only considerably longer survival on cocoa shells during storage (Komitopoulou and Peñaloza, 2009), but also greater thermal resistance during a thermal treatment (Keller et al., 2012). The inoculated cocoa beans used in the present study were stabilized for 16 h at 20°C to allow for adaptation of *Salmonella* cells to desiccation prior to roasting. This inoculum stabilization step may have improved the thermal resistance of *Salmonella* during roasting (Podolak et al., 2010; Peña-Meléndez et al., 2014; Pereira et al., 2020). Any or all of the above-outlined factors could have led to the increased thermal resistance of *Salmonella* and have resulted in higher *D_T_* values measured in the present study, as compared to the previous two studies (Izurieta and Komitopoulou, 2012; Nascimento et al., 2012). Our experimental procedure was designed to mimic not only the real-life but also worst-case scenario, where *Salmonella* is stabilized on cocoa beans during cocoa bean storage prior to roasting.

Although the use of *D* value is widely practiced, it still should be noted that microbial inactivation data may not always follow the first-order kinetics. For example, significant deviations from linearity were reported in past studies (Ma et al., 2009; Du et al., 2010). We hence analyzed all data using a Weibull model, in addition to the log-linear model (Figure 1). The SD of residuals of the Weibull model is shown as the RMSEs, which ranged from 0.16 to 0.39 log_10_ CFU/bean (Table 2). The *δ* values are considered as an alternative form of *D* values, which represent the time required to achieve the first decimal reduction, and they were generally lower compared to *D* values. The temperature dependence of *δ* was assessed by calculating as *z'* value, which was higher than the *z_T_* value (38.0 vs. 32.0°C). The values of the shape parameter *β* ranged from 0.52 to 0.99 (all <1), suggesting that inactivation rate of *S.* Oranienburg was decreasing over the roasting time (Figure 1). Our analysis also indicated that *β* did not appear to be dependent on the temperature (Table 2).

**Table 2:** Parameters of a log-linear model and a Weibull model describing the inactivation of *S.* Oranienburg strain FSL R9-6137 at different cocoa bean roasting temperatures.

Temperature (°C)	Log-linear model^a^				Weibull model^a^					
	*D*-value (min)	RSE (%)	RMSE (log CFU/bean)	*z_T_* (°C)	*δ* (min)	RSE (%)	*β*	RES (%)	RMSE (log CFU/bean)	*z'* (°C)
100	33.34	5.04	0.27	32.0 ± 1.6 (*R*^2^ = 0.99)	20.82	8.15	0.66	5.86	0.16	38.0 ± 3.9 (*R*^2^ = 0.84)
110	18.57	4.58	0.30		11.79	9.18	0.64	7.42	0.19	
115	12.92	4.15	0.52		4.30	29.95	0.52	13.35	0.36	
120	10.50	3.30	0.34		10.30	14.50	0.99	11.29	0.35	
130	4.20	1.70	0.20		3.60	7.60	0.90	4.98	0.19	
140	1.90	3.20	0.40		1.60	17.90	0.87	10.24	0.39	

a D-value, decimal reduction time.

Z_T_, increase in temperature that results in a reduction of a *D* value by a factor of 10; 𝛿, time required to achieve the first-log reduction at a given temperature; *β*, Weibull model shape parameter; *z'*, value for the Weibull model, which is analogous to the *z_T_* obtained using a log-linear model; RSE, relative standard error; RMSE, root mean square of residuals. A model’s goodness-of-fit was evaluated based on a RSE of estimated parameters and a RMSE of a model. *R*^2^ is a measure of goodness of fit of the secondary models.

In the present study, the deviation from a linear trend was observed when beans were roasted at 100, 110, and 115°C. Unlike the first-order kinetics where a constant rate of inactivation is assumed in the log-linear model, a decrease in the inactivation rate during roasting is indicated by the observed tailing phenomenon. It should be noted that the tailing phenomenon would likely be overlooked if roasting was conducted only within a narrow time range at these lower temperatures. The inactivation would, in that case, likely be overestimated if the model would have been extrapolated out of the experimentally tested range. To overcome this caveat, we experimentally tested a relatively wide range of roasting times (i.e., up to 100 min) at lower temperatures. The resulting observed tails could be attributed to the uneven heat distribution or changing water activity over time; however, factors related to cell physiology may likewise explain the presence of tails.

### Weibull Model Was a Better Fit to the *Salmonella* Oranienburg Thermal Inactivation Data Compared to the Log-Linear Model

In order to identify the model that could better explain the inactivation kinetics of *S.* Oranienburg during cocoa roasting, we compared the quality of the two models applied in this study. We calculated the AIC and BIC for each model (Table 1). Both AIC and BIC are penalized likelihood criteria used for model selection, and a lower value of AIC and BIC suggests a higher likelihood of model being true due to having a lower error. The Weibull models showed a significantly better fit to the *Salmonella* inactivation data obtained at 100, 110, 115, and 130°C (*p* < 0.05), while the goodness-of-fit of the two models was similar at 120 and 140°C, based on the comparison using ANOVA based on parsimony (Table 1). Weibull model was therefore identified as a more accurate model to describe *S.* Oranienburg thermal inactivation observed in this study. Furthermore, the underlying assumptions that make Weibull model more appropriate for describing the thermal inactivation of *S.* Oranienburg during cocoa roasting include the (1) variability in the intrinsic thermal resistance within a bacterial population, (2) microbial adaptation during heat treatment, and the (3) inactivation of multiple cellular components (Russell, 2003; Cebrián et al., 2017).

Although being generally a better fit, the application of Weibull models obtained in the present study is limited to the experimentally tested temperatures. This is due to the inability to predict the Weibull model shape parameter *β* at temperatures that had not been experimentally tested, whereas the calculation of the log-linear model *D* value allows for the inference of the *z_T_* value. As suggested by the low RMSEs (Table 2), the log-linear models were also a reasonably good fit to the *S.* Oranienburg inactivation data obtained in the present study. We therefore conclude that the log linear model is still useful for prediction of the inactivation kinetics of *S.* Oranienburg at temperatures that were not tested in the present study. Any predictions, however, are recommended to be used only as a guide for experimental validation. Finally, the limitations of this study include performing experiments on a single isolate of *S.* Oranienburg, recovering *Salmonella* colonies for just 24 h, performing experiments in conditions that did not allow for control of water activity, and using a non-full-factorial experimental design.

## Conclusion

We investigated the inactivation of *Salmonella* during cocoa roasting at a range of temperatures that are relevant to the production of fine chocolate. Our experimental results suggest that a reduction of >5 log_10_ CFU/bean can be achieved with 10-min roasting at 150°C. Our data analyses results suggested that a Weibull model more accurately models the thermal inactivation kinetics of *S.* Oranienburg strain used in this study, compared to the log-linear model. Using a Weibull model, the predicted times needed to achieve a 5-log_10_ reduction of *Salmonella* at temperatures of 100, 110, 115, 120, 130, and 140°C were 239, 146, 95, 53, and 10 min, respectively. The experimental results and models reported in this study may be used as a guide for the industry in-house validation of roasting processes using nonpathogenic surrogates of *Salmonella*, such as *Enterococcus faecium*.

## Data Availability Statement

The raw data supporting the conclusions of this article will be made available by the authors, without undue reservation.

## Author Contributions

RY conducted experiments and data analyses, and wrote the manuscript. GP conducted experiments and revised the manuscript. RT-R, KC, GD’A, and JK conceived the study and revised the manuscript. JK supervised the study and co-wrote the manuscript. All authors contributed to the article and approved the submitted version.

### Conflict of Interest

RT-R, KC, and GD’A were employed by the company Dandelion Chocolate Inc.

The remaining authors declare that the research was conducted in the absence of any commercial or financial relationships that could be construed as a potential conflict of interest.
