# Supplementary material for: Evaluation of the Thermal Inactivation of a Salmonella Serotype Oranienburg Strain During Cocoa Roasting at Conditions Relevant to the Fine Chocolate Industry
Source: Front Microbiol. 2021 Mar 8;12:576337. doi: 10.3389/fmicb.2021.576337 (PMC7982832; doi:10.3389/fmicb.2021.576337)
Supplement: Supplementary file 1 [file Table_1.docx]

Supplementary Material

**Evaluation of the thermal inactivation of a *Salmonella* serotype Oranienburg strain during cocoa roasting at conditions relevant to the fine chocolate industry**

Runan Yan^1^, Gabriella Pinto^1^, Rebecca Taylor-Roseman^2^, Karen Cogan^2^, Greg D’Alesandre^2^, Jasna Kovac^1^

^1^The Pennsylvania State University, Department of Food Science, Rodney A. Erickson Food Science Building, University Park, PA 16802, USA

^2^Dandelion Chocolate Inc., 740 Valencia Street, San Francisco, CA 94110, USA

**Supplementary Tables**

**Table S1.** Reduction of *Salmonella* due to cocoa roasting at different temperatures.

| Temperature (°C) | Time (min) | *Salmonella* (control, not roasted)^a^  (log_10_CFU/bean) | *Salmonella* (roasted)  (log_10_ CFU/bean)^a^ | Reduction of *Salmonella*  (log_10_ CFU/bean) | Average reduction of *Salmonella* ± standard error  (log_10_ CFU/bean) |
| --- | --- | --- | --- | --- | --- |
| 100 | 15 | 7.42 | 6.76 | 0.66 | 0.65 ± 0.13 |
|  |  | 7.4 | 6.72 | 0.67 |  |
|  |  | 7.36 | 6.59 | 0.77 |  |
|  |  | 6.9 | 6.44 | 0.45 |  |
|  |  | 6.83 | 6.29 | 0.54 |  |
|  |  | 7.03 | 6.25 | 0.78 |  |
|  | 20 | 7.23 | 5.82 | 1.42 | 1.29 ± 0.20 |
|  |  | 7.37 | 6.31 | 1.06 |  |
|  |  | 7.15 | 5.76 | 1.38 |  |
|  | 100 | 8.32 | 5.59 | 2.73 | 2.80 ± 0.09 |
|  |  | 8.58 | 5.68 | 2.9 |  |
|  |  | 8.12 | 5.34 | 2.78 |  |
| 110 | 15 | 7.39 | 6.23 | 1.15 | 1.34 ± 0.30 |
|  |  | 7.37 | 6.25 | 1.12 |  |
|  |  | 7.16 | 6.03 | 1.13 |  |
|  |  | 7.46 | 5.6 | 1.86 |  |
|  |  | 6.61 | 5.4 | 1.21 |  |
|  |  | 7.12 | 5.57 | 1.56 |  |
|  | 20 | 7.55 | 6.11 | 1.44 | 1.22 ± 0.29 |
|  |  | 7.41 | 6.5 | 0.91 |  |
|  |  | 7.53 | 6.53 | 1 |  |
|  |  | 7.31 | 5.69 | 1.62 |  |
|  |  | 7.26 | 5.91 | 1.35 |  |
|  |  | 6.51 | 5.5 | 1.01 |  |
|  | 60 | 8.65 | 5.73 | 2.93 | 2.87 ± 0.08 |
|  |  | 8.5 | 5.6 | 2.9 |  |
|  |  | 8.59 | 5.82 | 2.78 |  |
| 115 | 15 | 7.42 | 5.79 | 1.63 | 1.53± 0.56 |
|  |  | 7.46 | 5.43 | 2.03 |  |
|  |  | 7.15 | 6.22 | 0.93 |  |
|  | 20 | 7.35 | 4.36 | 2.99 | 2.16 ± 0.08 |
|  |  | 7.4 | 5.32 | 2.09 |  |
|  |  | 6.2 | 4.8 | 1.4 |  |
|  | 36 | 7.27 | 3.19 | 4.08 | 3.37 ± 0.44 |
|  |  | 7.37 | 3.84 | 3.53 |  |
|  |  | 7.15 | 4.36 | 2.79 |  |
|  |  | 7.4 | 4.35 | 3.06 |  |
|  |  | 7.26 | 3.97 | 3.29 |  |
|  |  | 7.34 | 3.88 | 3.46 |  |
|  | 45 | 8.61 | 5.03 | 3.58 | 3.66 ± 0.07 |
|  |  | 8.72 | 5.01 | 3.72 |  |
|  |  | 8.56 | 4.87 | 3.69 |  |
|  | 60 | 8.35 | 4.79 | 3.55 | 3.51 ± 0.05 |
|  |  | 8.55 | 5.03 | 3.52 |  |
|  |  | 8.48 | 5.03 | 3.46 |  |
| 120 | 15 | 8.43 | 6.72 | 1.71 | 1.37 ± 0.34 |
|  |  | 8.09 | 6.73 | 1.36 |  |
|  |  | 8.27 | 7.23 | 1.04 |  |
|  | 25 | 8.35 | 5.94 | 2.41 | 2.61 ± 0.34 |
|  |  | 8.25 | 5.83 | 2.43 |  |
|  |  | 8.37 | 5.37 | 3 |  |
|  | 35 | 8.05 | 5.08 | 2.97 | 3.12 ± 0.38 |
|  |  | 8.37 | 5.54 | 2.83 |  |
|  |  | 8.25 | 4.7 | 3.55 |  |
|  | 45 | 8.58 | 5.26 | 3.31 | 4.36 ± 0.91 |
|  |  | 8.4 | 3.55 | 4.85 |  |
|  |  | 8.31 | 3.4 | 4.91 |  |
| 130 | 2 | 8.47 | 7.6 | 0.87 | 0.75 ± 0.32 |
|  |  | 8.62 | 7.62 | 0.99 |  |
|  |  | 8.28 | 7.9 | 0.38 |  |
|  | 5 | 7.83 | 6.74 | 1.1 | 1.23 ± 0.18 |
|  |  | 8.12 | 6.77 | 1.36 |  |
|  | 10 | 8.3 | 5.76 | 2.54 | 2.36 ± 0.16 |
|  |  | 8.3 | 6.06 | 2.24 |  |
|  |  | 7.81 | 5.51 | 2.3 |  |
|  | 15 | 8.39 | 4.77 | 3.62 | 3.81 ± 0.38 |
|  |  | 8.24 | 4 | 4.24 |  |
|  |  | 8.35 | 4.79 | 3.56 |  |
|  | 20 | 8.3 | 3.57 | 4.73 | 4.62 ± 0.10 |
|  |  | 8.43 | 3.9 | 4.53 |  |
|  |  | 8.44 | 3.85 | 4.59 |  |
| 140 | 2 | 8.45 | 7.81 | 0.64 | 0.72 ± 0.17 |
|  |  | 8.31 | 7.41 | 0.91 |  |
|  |  | 8.19 | 7.58 | 0.61 |  |
|  | 5 | 7.48 | 4.46 | 3.03 | 3.32 ± 0.35 |
|  |  | 7.49 | 4.26 | 3.23 |  |
|  |  | 7.32 | 3.61 | 3.71 |  |
|  | 10 | 8.29 | 3.47 | 4.82 | 4.98 ± 0.51 |
|  |  | 8.17 | 3.28 | 4.89 |  |
|  |  | 8.25 | 3.44 | 4.81 |  |
|  |  | 8.26 | 2.41 | 5.85 |  |
|  |  | 8.06 | 3.55 | 4.52 |  |
| 150 | 2 | 8.28 | 6.93 | 1.35 | 1.42 ± 0.15 |
|  |  | 7.75 | 6.16 | 1.59 |  |
|  |  | 7.93 | 6.62 | 1.32 |  |
|  | 5 | 8.28 | 5.29 | 2.98 | 3.07 ± 0.29 |
|  |  | 8.41 | 5.58 | 2.84 |  |
|  |  | 8.27 | 4.88 | 3.4 |  |
|  | 10 | 8.37 | <1.3 | ND | >5 |
|  |  | 8.26 | < 1.3 | ND |  |
|  |  | 8.36 | < 1.3 | ND |  |

^a^ The values represent results for individual biological replicates calculated based on three technical replicates.
